# Research on Design Method of Multilayer Metamaterials Based on Stochastic Topology

**DOI:** 10.3390/ma16155229

**Published:** 2023-07-25

**Authors:** Zhipeng Xi, Xiaochi Lu, Tongsheng Shen, Chunrong Zou, Li Chen, Shaojun Guo

**Affiliations:** National Institute of Defense Technology Innovation, Academy of Military Sciences PLA China, Beijing 100171, China; xzp_paper@163.com (Z.X.);

**Keywords:** stochastic topology, metamaterial structure design, big data, automatic design

## Abstract

Metamaterials are usually designed using biomimetic technology based on natural biological characteristics or topology optimization based on prior knowledge. Although satisfactory results can be achieved to a certain extent, there are still many performance limitations. For overcoming the above limitations, this paper proposes a rapid metamaterials design method based on the generation of random topological patterns. This method realizes the combined big data simulation and structure optimization of structure-electromagnetic properties, which makes up for the shortcomings of traditional design methods. The electromagnetic properties of the proposed metamaterials are verified by experiments. The reflection coefficient of the designed absorbing metamaterial unit is all lower than −15 dB over 12–16 GHz. Compared with the metal floor, the radar cross section (RCS) of the designed metamaterial is reduced by a minimum of 14.5 dB and a maximum of 27.6 dB over the operating band. The performance parameters of metamaterial obtained based on the random topology design method are consistent with the simulation design results, which further verifies the reliability of the algorithm in this paper.

## 1. Introduction

Metamaterials have great application potential in the fields of stealth camouflage, electronic countermeasures, navigation and communication, early warning and guidance, and imaging recognition. The emergence of metamaterials provides a new idea for the design and preparation of absorbing materials. For a specific width of the frequency band, adjusting the artificial structural units of metamaterials can achieve effective modulation of the incident electromagnetic wave vector, thereby achieving nearly 100% perfect absorption characteristics. In 2008, Landy et al. [1,2,3,4,5] first proposed a theoretical model of a microwave-segment Metamaterial Absorber (MMA) and verified it experimentally. In the same year, Hu et al. [6]. realized MMA in the terahertz band by adjusting the structural parameters of MMA proposed by Landy et al. Through ingenious structural design, metamaterials can achieve a narrow-band electromagnetic wave absorption rate close to 100%, which has set off a wave of research on metamaterials [7,8,9,10,11]. Although early wave-absorbing metamaterials can achieve narrow-band and high-efficiency electromagnetic wave absorption [12,13,14,15,16,17,18], they neglected the insensitivity of electromagnetic absorbing materials to polarization direction [16,19,20,21,22,23,24], wide incident angle [25,26,27], multifrequency [18,28,29,30,31,32], broadband absorbing metamaterials [33,34,35,36], and electromagnetic parameters regulate metamaterials [35,36,37,38]. These are precisely the basis for the broadband and high-efficiency absorption of radar stealth metamaterials.

To solve the problem of polarization insensitivity, Cheng et al. [37] adopted a symmetric cross-resonant ring and continuous metal grid structure in 2011 to realize polarization-independent MMA. In 2012, Ding et al. [36] proposed a four-sided conical pyramid structure in the form of a metal-dielectric stack to meet the demand for broadband electromagnetic wave absorption in a special environment. In 2013, Yang et al. [38] designed an MMA with a high absorption rate and lossless layer. In 2014, Liu et al. [39] designed a Perfect Metametric Absorber (PMA), which greatly reduced the RCS of the antenna. In the same year, Zhu et al. [40] used a multilayer resonant composite structure to realize ultra-wideband microwave absorption. In 2015, Li et al. [41] designed scattered cross quasi-fractal structure materials to reduce the size. In 2020, Yang et al. [42] designed multilayer materials by compounding topological structures, which obtained a broadband low-scattering structure material.

At present, biomimetic technology that draws on the biological characteristics of nature or the topological optimization based on prior knowledge is usually used to design structural materials. There are still the following limitations: (1) Organisms in nature do not necessarily evolve in a way that provides the best structural properties. (2) It is difficult to find a completely corresponding organism from nature as a reference to obtain a matching biological structure. (3) The topology optimization method is restricted by the initial assignment of the structure. (4) Traditional design methods require rich design experience and expensive multiple experiments. Structural materials need to realize intelligent design. The machine can provide designers with more structural choices, provide powerful data support for the intelligent design of structural materials and specific performance, and adapt to complete the design of structural materials with specific performance and rapid calculation and evaluation of electromagnetic properties. According to the design characteristics of electromagnetic wideband absorbing structure materials, combined with the joint calculation method of Python and FDTD electromagnetic simulation, this paper proposes a method based on the automatic generation of random topologies and rapid transformation modeling and evaluation of structure-superstructure materials, so as to realize the structural design and optimization of electromagnetic absorbing superstructure materials with wide band and high absorption effect.

## 2. Microstructure Random Topology Generation Method

**Figure 1:**
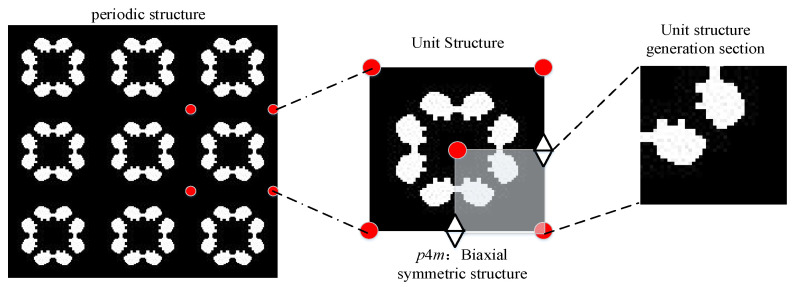
Schematic diagram of random topology generation.

From the perspective of electromagnetic wave broadband absorption, our work focuses on designing the generation method of the graphic structure. To obtain the topology of a structural material, we first generate the topology of its constituent elements (shown in Figure 1), map the elements to units of symmetry groups, and then periodically switch the units to form the overall structural material. It is worth noting that for a systematic design method, the topology of the graphic elements needs to meet the following conditions: (1) The topology should be randomly generated to represent the entire design space; (2) The number of pixels of a complete graphic should follow the specified structure ratio; (3) The structure blocks in the graphic unit need to be connected.

**Figure 2:**
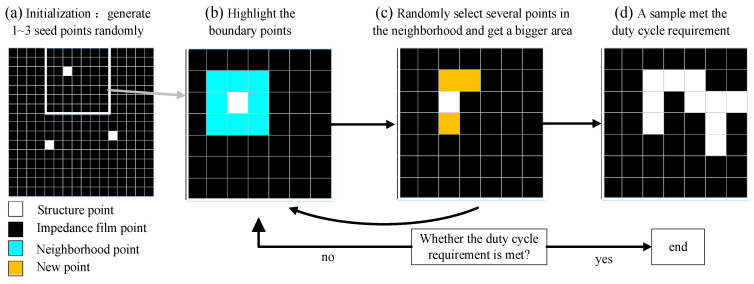
Schematic diagram of the generation process of microstructure random topological growth. (**a**) Initialize and randomly generate seed points; (**b**) Mark the field pixels of the seed points; (**c**) Randomly select several points from the field as new structure points; (**d**) Obtain the region structure that meets the duty cycle requirements.

To generate the desired configuration of graphic elements, random generation and symmetry algorithms are developed, and the microstructure generation process is shown in Figure 2. The size of each pixel is sent as 0.1 × 0.1 mm^2^ in this paper. Firstly, randomly generate a number of structural pixel seed points in the agreed area. Secondly, starting from all structural pixels, calculate and mark the neighborhood boundary. Thirdly, further randomly select a number of pixels from the boundary pixel coordinates that meet the constraints as new structural pixels. Meanwhile, one to three point pixels are randomly assigned as new structure seed points, which is beneficial to the structure generation with high structure pixel ratio. In addition, during the test, it is found that if the addition of new structural seed points is not considered, the growth rate would be too slow in the process of graph generation limited by some special regions, resulting in low graph generation efficiency.

**Figure 3:**
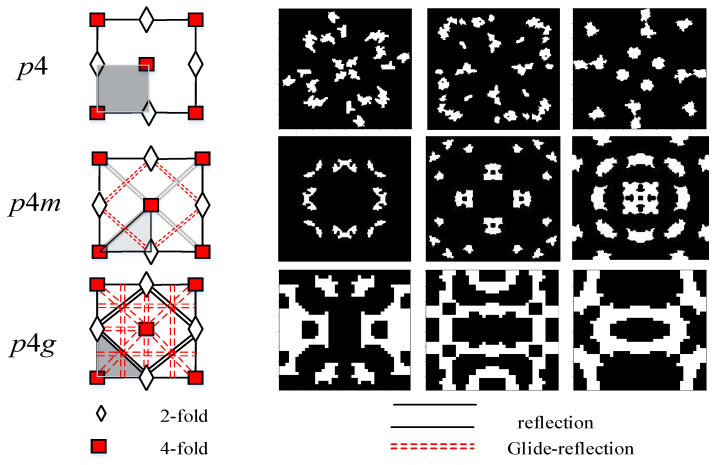
Microstructure Generation Mode of Electromagnetic Absorbing Metamaterials.

For electromagnetic wave absorbing stealth, the polarization direction of the incident electromagnetic wave and the relative angular position of the material have a great influence on the absorbing effect. In order to adapt to the change of polarization direction as much as possible, this paper adopts the method of rotation and axis symmetry in the process of graph generation. A variety of topological modes, according to this principle, can generate any number of partitioned graphics in principle to achieve the consistency of the horizontal and vertical polarization of electromagnetic wave absorption. The generation mode used in this paper is shown in Figure 3. Among them, p4 adopts the rotation generation mode for structure generation, and p4m adopts the axisymmetric mode for generation. These two generation modes have good polarization adaptability, and p4g adopts uniaxial symmetry. The unit structure is sensitive to electromagnetic wave polarization, but the metamaterial can be realized with strong polarization adaptability by symmetrical combination over a large area.

**Figure 4:**
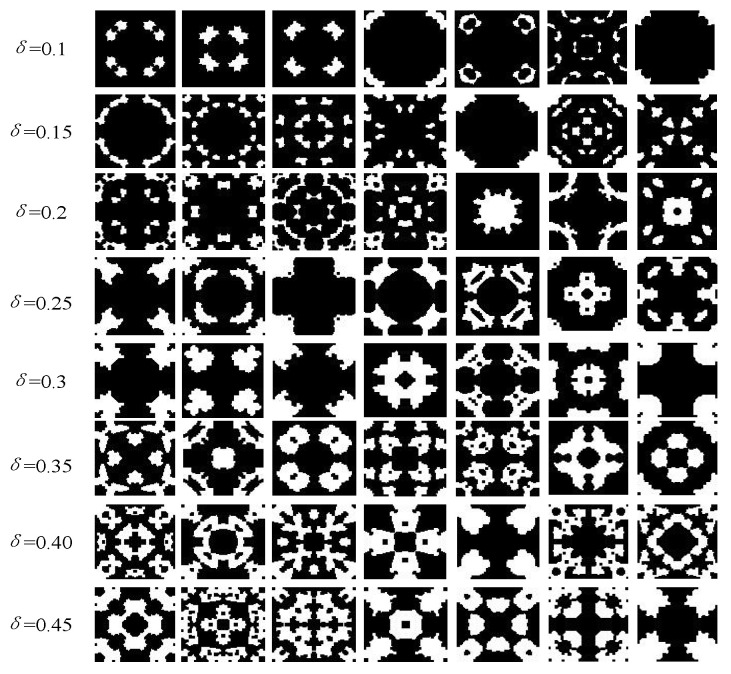
Different *δ* biaxially symmetric polarization-insensitive microstructures.

The microstructure generation algorithm can generate different structures according to the requirements of different structural duty ratios. Based on determining the optimal duty cycle, simulation calculations and performance evaluations for a large number of structural materials can be performed, which can realize the rapid development of high-efficiency electromagnetic wave-absorbing metasurfaces. For optimization, Figure 4 presents microstructure samples with different structural duty cycles *δ*. The intelligent generation and automated test evaluation of massive microstructures is an effective approach for designers.

## 3. Electromagnetic Material Generation and Field Combined Testing

Combining with the microstructure generation mode in Figure 3, the structural characteristics of the material can be obtained. For the corresponding structural elements, the electromagnetic parameters of the material need to be further assigned, and different material systems can be generated according to different needs. In the electromagnetic wave absorbing material system, the electromagnetic microstructure itself cannot realize the wave absorbing function alone, and it needs to be multilayered with the substrate dielectric material to achieve the RCS reduction effect. This paper also explores the electromagnetic wave absorbing properties of single-layer microstructured material and dielectric composite and multilayer microstructured film and multilayer dielectric composite metamaterial, and realizes microstructure encoding storage and metamaterial electromagnetic reflection properties through Python and FDTD. Big data intelligent testing and storage of S-parameters are considered. During the storage process, the corresponding data file implementation structure is constructed with a one-to-one mapping of S-parameters.

**Figure 5:**
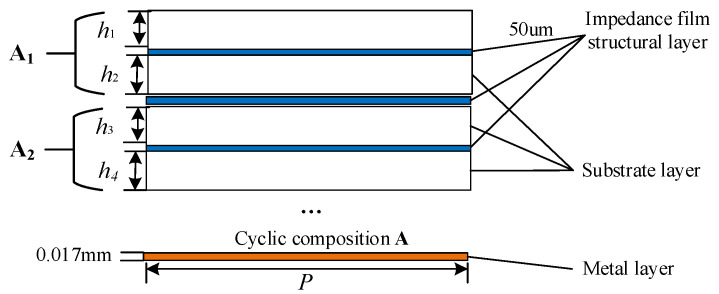
Multilayer electromagnetic wave-absorbing metamaterial composite method. A_1_ consists of two lower dielectric layers and an intermediate impedance layer, which can be repeated many times in the Z direction, *h*_1_ and *h*_2_ are the thickness of the upper and lower dielectric layers, respectively. A_2_ is similar to A_1_, the thickness of the upper and lower layers of A_2_ medium *h*_3_ and *h*_4_ can be designed according to the needs, and the microstructure of the intermediate impedance layer of A_2_ can also be designed according to the needs.

The generation method of metamaterials containing microstructures is shown in Figure 5. Since the impedance film is easily damaged, the electromagnetic performance is degraded. In this paper, the environment-resistant medium is covered on the surface layer, and the impedance structure layer is loaded in the middle of the medium. Composite processing, as shown in the composite schematic diagram of the multilayered microstructured material in Figure 5, occurs where the microstructures are randomly selected when repeating the formation of A.

Taking the search for high-efficiency broadband absorbing metamaterials in the 12–16 GHz frequency band as an example, set the microstructure period *P* = 8 mm, the impedance film is polyimide film, the impedance is 95 Ohm/sq, the medium is cyanate ester, its dielectric constant is 3.0 and the loss tangent is 0.005. The upper and lower layers of dielectrics are selected to have the same thickness to achieve resonance characteristics in the frequency band and increase the absorption effect. The theoretical Formula (1) is used to calculate the thickness [43].

(1) $$d=\lambda /4\sqrt{\varepsilon_{sub}}=c/4f\sqrt{\varepsilon_{sub}}$$

**Figure 6:**
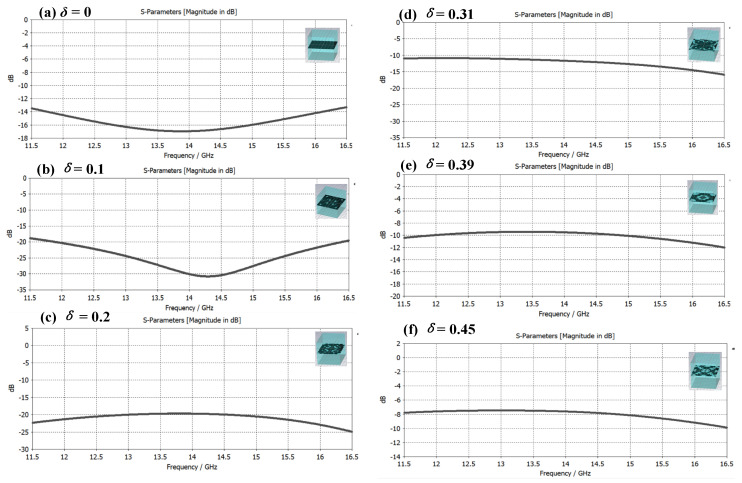
Metamaterial samples with different structural duty ratios and their electromagnetic reflection S-parameter characteristic curves in the range of 12–16 GHz. (**a**) Electromagnetic reflection characteristics of dielectric-impedance film-dielectric composite metamaterials without microstructures; (**b**–**f**) Electromagnetic absorption characteristics of microstructures with different structural duty ratios, where *δ* is 0.1 and 0.15, the electromagnetic absorption performance is greatly improved, and the *δ* continues to increase, but the performance decreases.

Among them, *d* is the resonance thickness of the electromagnetic wave, *c* is the propagation speed of the electromagnetic wave, *f* is the frequency of the electromagnetic wave, ε is the dielectric constant of the medium, and λ is the wavelength of the microwave. Through calculation, the corresponding thicknesses of the 12 GHz and 16 GHz frequency bands are 3.6 mm and 2.7 mm, respectively. The thickness is weighed under the condition of wide frequency and *d* = 3.1 mm ($\approx \sqrt{3.6\times 2.7}$) is selected with reference to Formula (2). Figure 6 represents a graph showing the composite effect of metamaterials with a single-layer impedance film and different structural duty ratios *δ* and their electromagnetic reflection S-parameter characteristic curves in the frequency band of 12–16 GHz. Since the bottom layer of the metasurface is a layer of metal flakes, which can realize the total reflection of electromagnetic waves, the electromagnetic reflection S-parameter curve directly reflects the electromagnetic absorption characteristics of the metasurface material. The smaller the reflection S-parameter, the better the electromagnetic wave absorption effect. From the simulation calculation results in Figure 6, it can be found that the material composite mode of dielectric-impedance film-dielectric has a certain electromagnetic absorption effect when there is no microstructure. After that, the electromagnetic absorption performance of the material is obviously enhanced, the structure duty ratio *δ* continues to increase, and the electromagnetic absorption effect gradually weakens.

(2) $$d_{multi-band}=\sqrt{d_{f\min}\cdot d_{f\max}}$$

**Figure 7:**
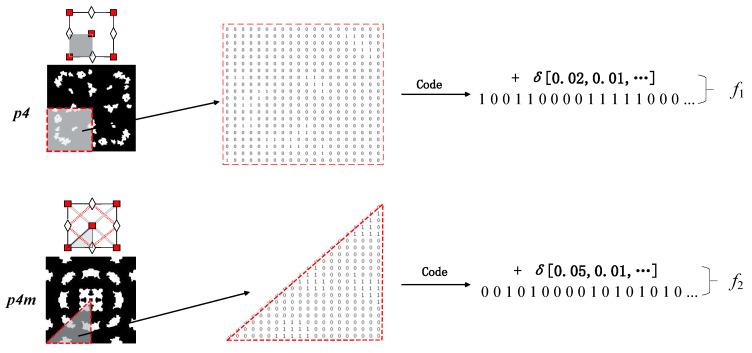
Metamaterial Coding Method.

In order to liberate the workload of researchers, this paper uses Python to generate electromagnetic microstructure patterns, converts the patterns into GDS files that can be called by FDTD, uses the interface between Python and FDTD to import GDS microstructures, and then controls the generation of superstructures according to the method in Figure 5. The material is constructed and the electromagnetic performance simulation test is carried out. After the simulation calculation is completed, the automatic storage algorithm will encode and save the microstructure (the encoding method is shown in Figure 7), and at the same time save the electromagnetic reflection S-parameter data and phase data of the metamaterial in the same index position in the corresponding data file, which is convenient for subsequent calculations.

## 4. Simulation and Analysis

**Figure 8:**
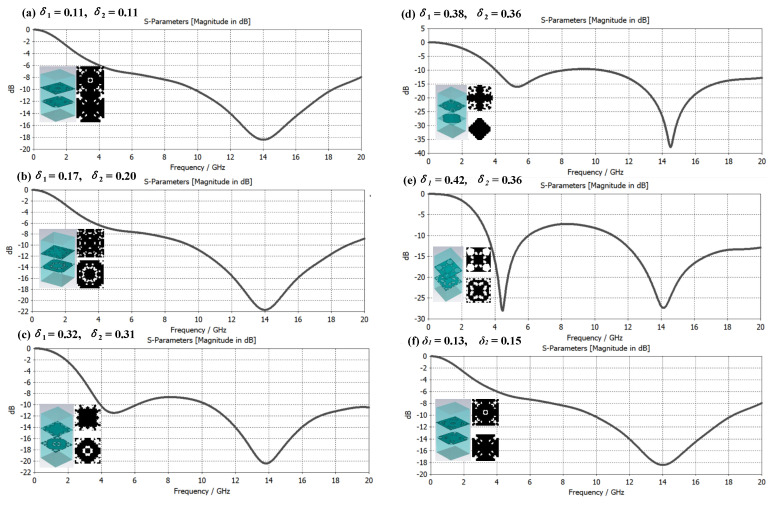
Case ① Metamaterial broadband electromagnetic absorption characteristic curve under the condition of *δ*_1_ *≈ δ*_2_.

**Figure 9:**
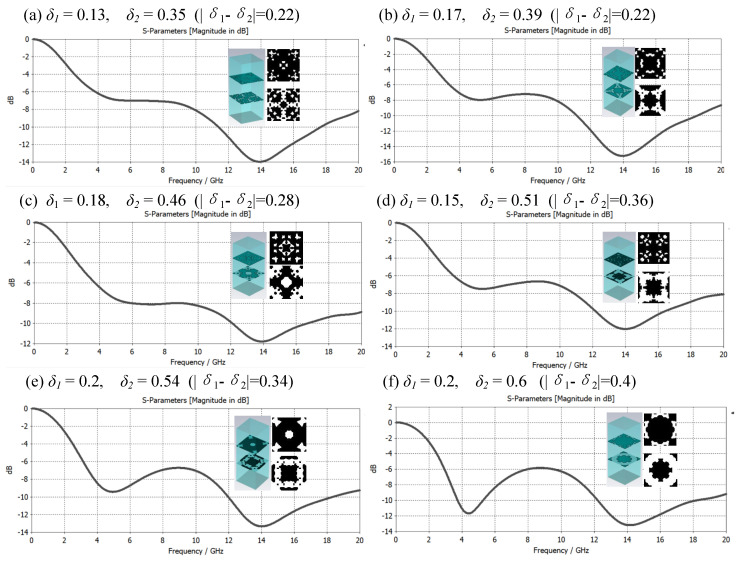
Case ② Metamaterial broadband electromagnetic compatibility absorption characteristic curve under the condition of *δ*_1_ < *δ*_2_.

**Figure 10:**
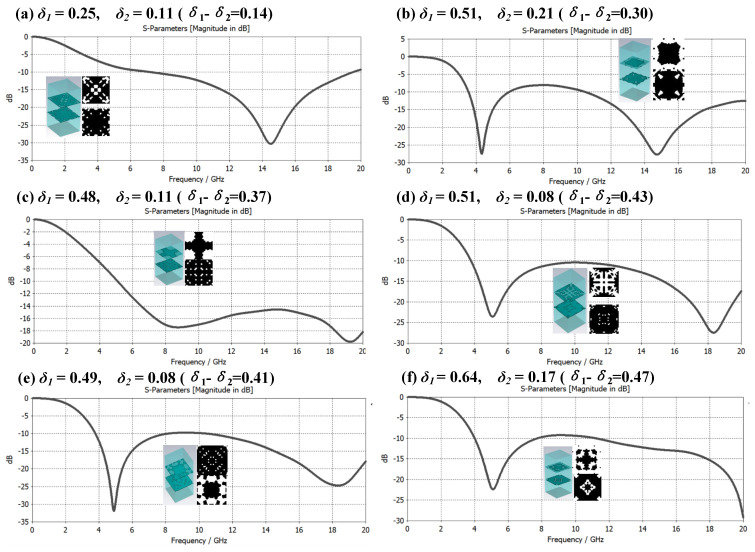
Case ③ Metamaterial broadband electromagnetic compatibility absorption characteristic curve under the condition of *δ*_1_ > *δ*_2_.

The composite of single-layer microstructure and medium can obtain a better electromagnetic wave absorption effect in a certain frequency band. Can multilayered microstructure and medium composite achieve better electromagnetic wave absorption effect, or a larger bandwidth compatible absorption? Based on this consideration, this paper constructs a double-layer microstructure metamaterial based on the δ value of the structural duty cycle and conducts an electromagnetic performance simulation analysis. There are three cases as follows: ① *δ*_1_ ≈ *δ*_2_; ② *δ*_1_ < *δ*_2_; ③ *δ*_1_ > *δ*_2_. Figure 8, Figure 9 and Figure 10 are the samples of double-layer microstructure metamaterials and their electromagnetic wave absorption effects in the frequency range of 0 to 20 GHz under the above three cases, respectively. The microstructure period is set to *P* = 8 mm, and the thickness of each layer is the same at 3.1 mm, and the material properties are consistent with those of the metamaterial in Figure 6.

As shown in Figure 8, it is a graph of the broadband electromagnetic compatibility absorption characteristic curve of metamaterials under the condition ① *δ*_1_ ≈ *δ*_2_, and the structure duty cycle value changes from small to large. Through research, it was found that the electromagnetic wave absorption characteristics did not change significantly in the frequency band of 12–20 GHz, and the main resonance frequency was located near 14 GHz. It is worth noting that in the frequency band below 12 GHz, the electromagnetic absorption characteristics are greatly affected by the values of *δ*_1_ and *δ*_2_, and when case ② *δ*_1_ is slightly larger than *δ*_2_, the electromagnetic absorption effect is better than when *δ*_2_ is slightly larger than *δ*_1_, and the resonance frequency is located at 4.5 GHz. Nearby, when the resonance frequency absorption effect is very good, the broadband compatibility of the metamaterials is often poor.

The double-layer microstructure pattern of the metamaterial and the absorption characteristic curve of broadband electromagnetic compatibility are shown in Figure 9. The simulation results show that when the value of |*δ*_1_ − *δ*_2_| increases, the electromagnetic absorption effect of metamaterials in the low-frequency region (6–10 GHz) will be enhanced, but in the high-frequency region (12–20 GHz)) it will be less effective. This shows that in the range of 0–20 GHz, the large-bandwidth electromagnetic compatibility absorption effect of the metamaterial designed in this mode is not obvious. Therefore, the structure-S-parameter big data of the mode of structural duty ratio *δ*_1_ *< δ*_2_ can be excluded. This is generated to save the cost of optimization calculation.

From the simulation results shown in Figure 10, it can be found that under the condition of ③ *δ*_1_ > *δ*_2_, when the difference between the duty ratios of the upper and lower microstructures *δ*_1_ − *δ*_2_ increases from small to large, the broadband electromagnetic compatibility absorption of metamaterials effect is enhanced. If the value of *δ*_1_ − *δ*_2_ continues to increase, the electromagnetic absorption capacity of the middle frequency band will become weaker. It is found from the figure that the difference is about 0.37. Through the structural optimization search, all electromagnetic waves in the 6–18 GHz frequency band can be absorbed by more than 10 dB (see Figure 10c). Through the study of the above three cases, a metamaterial composite mode with high-efficiency electromagnetic compatibility absorption characteristics with large bandwidth can be obtained, which can guide the generation and storage of structure-S-parameter big data, complete the optimization of the microstructure, and save calculation and storage costs.

**Figure 11:**
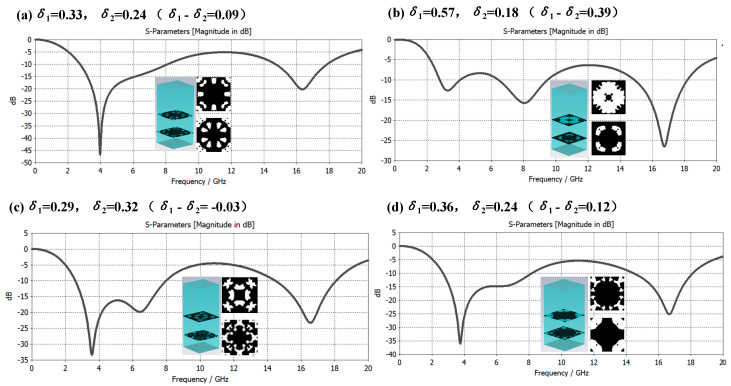
Broadband Absorption Effect of Different Microstructures with 7.9–4.6–3.0 mm Dielectric Thickness.

In the above research, the thickness of each layer of the metamaterial is the same. Can changing the thickness of different layers achieve better broadband electromagnetic compatibility absorption performance? In order to explore this problem, under the same material selection system metamaterials with different thicknesses are constructed and new constraints are proposed. That is, the upper layer medium is mainly used to resonate in the 4–8 GHz frequency band and the middle layer medium is mainly used to resonate in the 8–12 GHz frequency band. In the 12 GHz band region, the lower dielectric mainly resonates in the 12–18 GHz band region. According to Formula (1), the thickness of the dielectric layer from top to bottom can be calculated to be 7.9 mm, 4.6 mm, and 3.0 mm, respectively, while the total thickness is 15.5 mm, as shown in the Figure 11 which is a graph of broadband electromagnetic wave absorption characteristics corresponding to different microstructured composite metamaterials.

According to Formula (1), it can be obtained that the resonant frequencies corresponding to the dielectric layers with thicknesses of 7.9 mm, 4.6 mm, and 3.0 mm should be around 5.6 GHz, 10 GHz, and 15 GHz, respectively. However, it can be found from the curves in Figure 11 that the microstructures with different structural duty cycle δ values will cause different degrees of red shift of the two design resonant frequencies that should appear at 5.6 GHz and 10 GHz. The resonance frequency that appears at 15 GHz has a certain degree of blue shift. Therefore, the thickness of the dielectric layer needs to be optimized. This characteristic of the microstructure provides the possibility of reducing the overall thickness of the metamaterial, making it so that the metamaterial practicability is further improved.

## 5. Big Data Structure Design and Optimization

The above studies show that for single-layer structures, only microstructure optimization is needed, while for multilayer structures, it needs to be divided into two modes: optimization under the condition of fixed dielectric layer thickness and optimization of multilayer thickness and microstructure [43]. This paper mainly studies the optimization process under the conditions of multilayer thickness and different microstructures, but the optimization methods in other situations are also similar. Taking the double-layer microstructure composite three-layer dielectric metamaterial as an example, it is necessary to determine the thickness distribution of the dielectric layers that meet the conditions. Adjust the thickness of different dielectric layers to achieve various combinations and optimize the selection of thickness matching that meets the absorption index to achieve large-bandwidth electromagnetic compatibility absorption effects, so as to achieve the goal of better than −10 dB in the 6–18 GHz frequency band. The evaluation criteria are shown in the Formula (3).

(3) $$F_{score}\;=\;\min(\mathrm{avg}\sum_{f\;=\;f_{\min}}^{f_{\max}}\left(S_{f}\;-\;(-10\mathrm{dB})\right))\;@\;S_{f}\;\leq \;-10\mathrm{dB},\;f\in (6,18)\;\mathrm{GHz}$$

**Figure 12:**
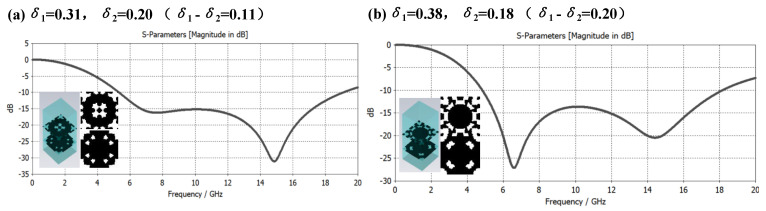
Broadband electromagnetic compatibility wave absorption characteristics of metamaterials combined with two microstructures when the thickness of the dielectric layer is 3.6–2.6–1.8 mm.

In this paper, two different microstructure combinations are selected, and the simulation results that meet the requirements after optimizing the thickness are shown in Figure 12. The two different structure combinations meet the index requirements when the thickness of the three-layer dielectric is 3.6 mm, 2.6 mm, and 1.8 mm, respectively. However, differences in the microstructure will cause differences in the compatible wave absorbing ability, so it is necessary to carry out the second stage of optimization which is microstructure optimization.

In the process of optimization, in order to avoid the miss-selecting of metamaterials with poor electromagnetic compatibility absorption performance but large absorption troughs, Formula (3) can be improved, and the evaluation of compatible absorption efficiency is carried out by means of subsection evaluation and weighting. (See Equation (4)).

(4) $$\begin{array}{l} F_{score}=\min(a_{1}\times \mathrm{avg}\sum_{f=f_{1}}^{f_{2}}\left(S_{f}-(-10\mathrm{dB})\right)+a_{2}\times \mathrm{avg}\sum_{f=f_{2}}^{f_{3}}\left(S_{f}-(-10\mathrm{dB})\right)+a_{3}\times \mathrm{avg}\sum_{f=f_{3}}^{f_{4}}\left(S_{f}-(-10\mathrm{dB})\right))\;\\ \;@\;S_{f}\leq -10\mathrm{dB},\;f\in (6,18)\mathrm{GHz},\;f_{1}=6\mathrm{GHz},\;f_{2}=10\mathrm{GHz},\;f_{3}=14\mathrm{GHz},\;f_{4}=18\mathrm{GHz} \end{array}$$

Among them, *a*_1_, *a*_2_, and *a*_3_ are the weights of the average values of the three frequency bands, which can be adjusted according to different needs.

**Figure 13:**
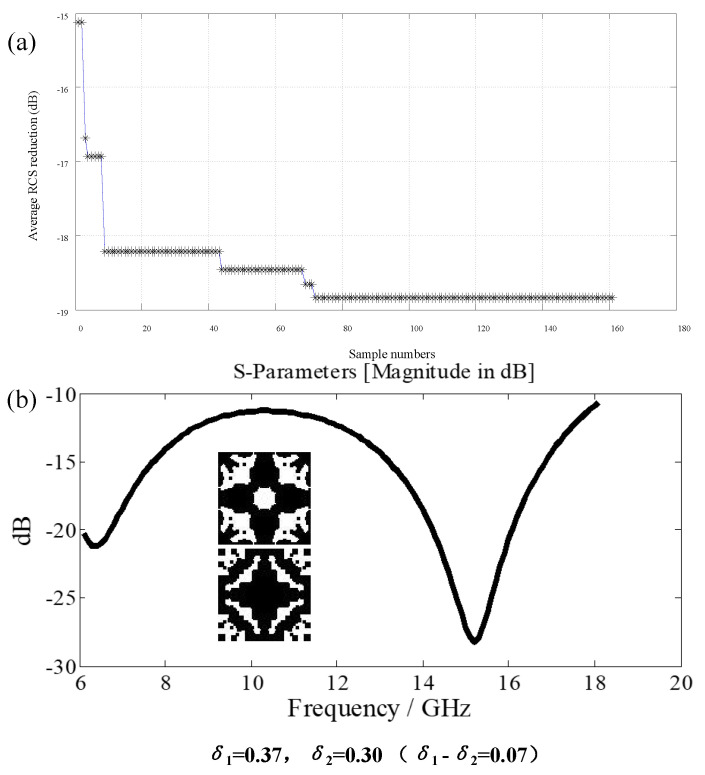
(**a**) Convergence curve of big data optimization; (**b**) S—scattering curve of optimized structure.

Taking 3.6 mm, 2.6 mm, and 1.8 mm for the thickness of the three-layer medium as constraints, a big data optimization is conducted. The random *δ*_1_ and *δ*_2_ combinations are used in the optimization process. After 160 iterations of optimization, the convergence curve is shown in Figure 13. In this paper, the evaluation criterion uses Formula (3) to calculate the score and combines the random process for optimization so there is a certain randomness.

## 6. Experimental Verification and Conclusions

**Figure 14:**
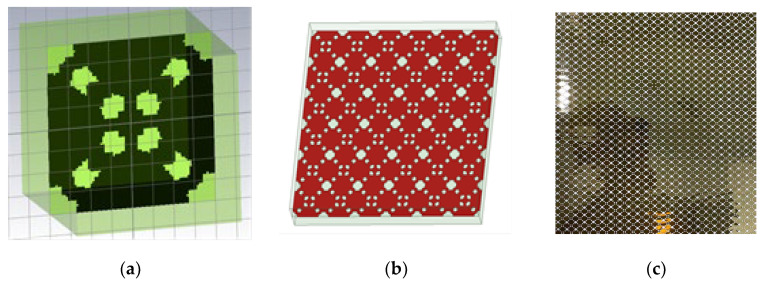
Optimize the structure and metamaterial plate. (**a**) Meta structure; (**b**) periodic structure; (**c**) Process physical objects.

**Figure 15:**
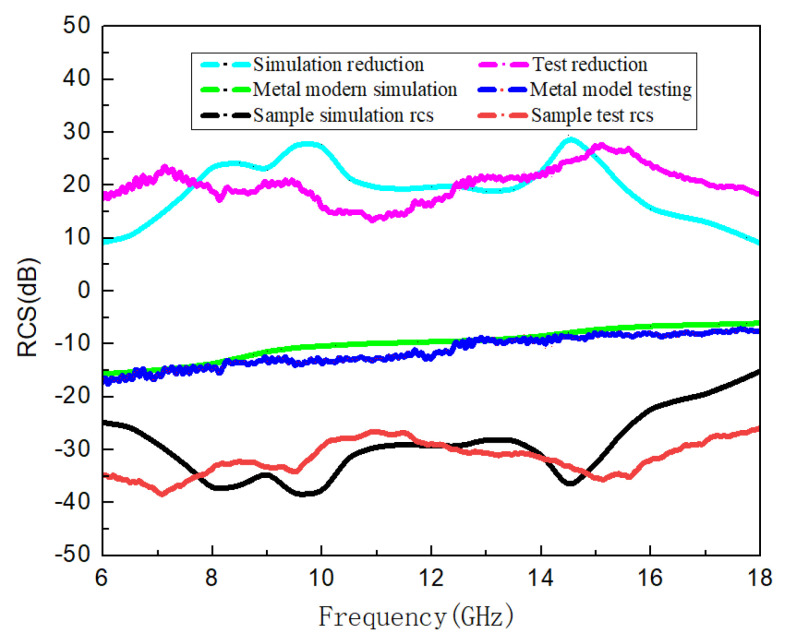
Comparison of RCS reduction simulation and measured results.

In order to further verify the applicability of the algorithm presented in this paper, we set the total thickness of metamaterials to 7.4 mm, the microstructure layer is a single-layer highly absorbing metamaterial in the 8–16 GHz frequency band, and the upper and lower dielectric layers are of equal thickness. After designing and optimizing the structure, the structure of the metamaterial is obtained and the solid sample is prepared. The results are shown in Figure 14. It is found that there is a certain deviation between the measured reflection S-parameter curve of the solid sample and the result simulated by the FDTD method (see Figure 15), which may be caused by a machining accuracy error during the processing of the sample. The microstructure error obtained by the design method proposed in this paper has high requirements on the processing technology, which often causes the deviation of the design index and the test index of the processed sample. This is also a possible deficiency of the algorithm in this paper, but it does not affect the applicability of the design method.

In summary, the design method of structural materials based on random topology proposed in this paper can automatically and quickly generate multiple styles of structural materials, and the electromagnetic characteristics of structural materials can be quickly realized by a combining FDTD algorithm. Taking 12–16 GHz high efficiency absorption as an example, a structure is selected and tested by mass structure generation and performance evaluation before the reliability of the design method is verified. The main purpose of this paper is to study the method of automatic structural design, which can be extended to other structural material design fields with the needs of other bands and electromagnetic properties.

## Data Availability

No new database has been created, please contact the author if required.

## References

[1] Teyssier J., Saenko S.V., Van Der Marel D., Milinkovitch M.C. (2015). Photonic crystals cause active color change in chameleons. Nat. Commun..

[2] Smith D.R., Padilla W.J., Vier D.C., Nemat-Nasser S.C., Schultz S. (2000). Composite medium with simultaneously negative permeability and permittivity. Phys. Rev. Lett..

[3] Shelby R.A., Smith D.R., Schultz S. (2001). Experimental verification of a negative index of refraction. Science.

[4] Pendry J.B. (2000). Negative refraction makes a perfect lens. Phys. Rev. Lett..

[5] Landy N.I., Sajuyigbe S., Mock J.J., Smith D.R., Padilla W.J. (2008). Perfect metamaterial absorber. Phys. Rev. Lett..

[6] Tao H., Landy N.I., Bingham C.M., Zhang X., Averitt R.D., Padilla W.J. (2008). A metamaterial absorber for the terahertz regime: Design, fabrication and characterization. Opt. Express.

[7] Ji D., Song H., Zeng X., Hu H., Liu K., Zhang N., Gan Q. (2014). Broad band absorption engineering of hyperbolic meta film patterns. Sci. Rep..

[8] Zhong S., He S. (2013). Ultrathin and light weight microwave absorbers made of mu-near-zero metamaterials. Sci. Rep..

[9] Wattsc M., Liu X., Padilla W.J. (2012). Metamaterial electro-magnetic wave absorbers. Adv. Mater..

[10] Sun J., Liu L., Dong G., Zhou J. (2011). An extremely broadband metamaterial absorber based on destructive interference. Opt. Express.

[11] Narimanov E.E., Kildishev A.V. (2009). Optical black hole: Broad band omnidirectional light absorber. Appl. Phys. Lett..

[12] Liu X., Zhao Q., Lan C., Zhou J. (2013). Isotropic Mie resonance-based metamaterial perfect absorber. Appl. Phys. Lett..

[13] Jiang Z.H., Yun S., Toor F., Werner D.H., Mayer T.S. (2011). Conformal dual-band near-perfectly absorbing mid-infrared metamaterial coating. ACS Nano.

[14] Hao J., Wang J., Liu X., Padilla W.J., Zhou L., Qiu M. (2010). High performance optical absorber based on a plasmonic metamaterial. Appl. Phys. Lett..

[15] Cui Y., Fung K.H., Xu J., Ma H., Jin Y., He S., Fang N.X. (2012). Ultrabroad band light absorption by a sawtooth anisotropic metamaterial slab. Nano Lett..

[16] Cheng Q., Cui T.J., Jiang W.X., Cai B.G. (2010). An omnidirectional electromagnetic absorber made of metamaterials. New J. Phys..

[17] Cao T., Wei C.W., Simpson R.E., Zhang L., Cryan M.J. (2014). Broad band polarization-independent perfect absorber using a phase-change metamaterial at visible frequencies. Sci. Rep..

[18] Liu X., Lan C., Bi K., Li B., Zhao Q., Zhou J. (2016). Dual band metamaterial perfect absorber based on Mie resonances. Appl. Phys. Lett..

[19] Grant J., Ma Y., Saha S., Lok L.B., Khalid A., Cumming D.R. (2011). Polarization insensitive terahertz metamaterial absorber. Opt. Lett..

[20] Grant J., Ma Y., Saha S., Khalid A., Cumming D.R. (2011). Polarization in sensitive, broad band terahertz metamaterial absorber. Opt. Lett..

[21] Huang X., Zhang C., Cong L., Fan J., Yuan H. (2011). Polarization insensitive and omnidirectional broadband near perfect planar metamaterial absorber in the near infrared regime. Appl. Phys. Lett..

[22] Aydin K., Ferry V.E., Briggs R.M., Atwater H.A. (2011). Broadband polarization-independent resonant light absorption using ultrathin plasmonic super absorbers. Nat. Commun..

[23] Zhu B., Wang Z., Huang C., Feng Y., Zhao J., Jiang T. (2010). Polarization insensitive metamaterial absorber with wide incident angle. Prog. Electromagn. Res..

[24] Landy N.I., Bingham C.M., Tyler T., Jokerst N., Smith D.R., Padilla W.J. (2009). Design, theory, and measurement of a polarization-insensitive absorber for terahertz imaging. Phys. Rev. B.

[25] Shen X., Cui T.J., Zhao J., Ma H.F., Jiang W.X., Li H. (2011). Polarization-independent wide-angle triple-band metamaterial absorber. Opt. Express.

[26] Pu M., Hu C., Wang M., Huang C., Zhao Z., Wang C., Feng Q., Luo X. (2011). Design principles for infrared wide-angle perfect absorber based on plasmonic structure. Opt. Express.

[27] Li L., Yang Y., Liang C. (2011). A wide-angle polarization-insensitive ultra-thin metamaterial absorber with three resonant modes. J. Appl. Phys..

[28] Park J.W., Van Tuong P., Rhee J.Y., Kim K.W., Jang W.H., Choi E.H., Chen L.Y., Lee Y. (2013). Multiband metamaterial absorber based on the arrangement of donut-type resonators. Opt. Express.

[29] Huang X., Yang H., Yu S., Wang J., Li M., Ye Q. (2013). Triple band polarization insensitive wide angle ultra-thin planar spiral metamaterial absorber. J. Appl. Phys..

[30] Xu H.X., Wang G.M., Qi M.Q., Liang J.G., Gong J.Q., Xu Z.M. (2012). Triple-band polarization insensitive wide-angle ultra-miniature metamaterial transmission line absorber. Phys. Rev. B.

[31] Shen X., Yang Y., Zang Y., Gu J., Han J., Zhang W., Jun Cui T. (2012). Triple-band terahertz metamaterial absorber: Design, experiment, and physical inter pretation. Appl. Phys. Lett..

[32] Liu X., Lan C., Li B., Zhao Q., Zhou J. (2016). Dual band metamaterial perfect absorber based on artificial dielectric “molecules”. Sci. Rep..

[33] Wang B.X., Wang L.L., Wang G.Z., Huang W.Q., Li X.F., Zhai X. (2014). Theoretical investigation of broad band and wide-angle terahertz metamaterial absorber. IEEE Photonics Technol. Lett..

[34] Wu C., Shvets G. (2012). Design of metamaterial surfaces with broad band absorbance. Opt. Lett..

[35] Huang L., Chowdhury D.R., Ramani S., Reiten M.T., Luo S.N., Taylor A.J., Chen H.T. (2012). Experimental demonstration of terahertz metamaterial absorbers with a broad and flat high absorption band. Opt. Lett..

[36] Ding F., Cui Y., Ge X., Jin Y., He S. (2012). Ultra-broadband microwave metamaterial absorber. Appl. Phys. Lett..

[37] Cheng Y., Yang H., Cheng Z., Wu N. (2011). Perfect metamaterial absorber based on a split-ring-cross resonator. Appl. Phys. A.

[38] Yang H., Cao X., Gao J., Liu T., Ma J., Yao X., Li W. (2013). Design of microstrip antenna with low radar cross section based on ultrasonic absorber. Acta Phys. Sin..

[39] Liu Y., Zhao X. (2014). Perfect Absorber Metamaterial for Designing Low-RCS Patch Antenna. IEEE Antennas Wirel. Propag. Lett..

[40] Zhu J., Ma Z., Sun W., Ding F., He Q., Zhou L., Ma Y. (2014). Ultra-broadband terahertz metamaterial absorber. Appl. Phys. Lett..

[41] Zhou Z., Chen K., Zhu B., Zhao J., Feng Y., Li Y. (2018). Ultra-wideband Microwave Absorption by Design and Optimization of Metasurface Salisbury Screen. IEEE Access.

[42] Yang J., Huang C., Song J., Ji C., Luo X. (2020). Ultra-broadband low scattering meta surface utilizing mixed-elements based on phase cancellation. J. Phys. D Appl. Phys..

[43] de Araújo J.B., Siqueira G.L., Kemptner E., Weber M., Junqueira C., Mosso M.M. (2020). An Ultrathin and Ultrawideband Meta material Absorber and an Equivalent-Circuit Parameter Retrieval Method. IEEE Trans. Antennas Propag..

